# Hierarchical Bayesian modelling of gene expression time series across irregularly sampled replicates and clusters

**DOI:** 10.1186/1471-2105-14-252

**Published:** 2013-08-20

**Authors:** James Hensman, Neil D Lawrence, Magnus Rattray

**Affiliations:** 1 Department of Computer Science, The University of Sheffield, Sheffield, UK; 2 Department of Neuroscience, The University of Sheffield, Sheffield, UK; 3 Faculty of Life Science, The University of Manchester, Manchester, UK

## Abstract

**Background:**

Time course data from microarrays and high-throughput sequencing experiments require simple, computationally efficient and powerful statistical models to extract meaningful biological signal, and for tasks such as data fusion and clustering. Existing methodologies fail to capture either the temporal or replicated nature of the experiments, and often impose constraints on the data collection process, such as regularly spaced samples, or similar sampling schema across replications.

**Results:**

We propose hierarchical Gaussian processes as a general model of gene expression time-series, with application to a variety of problems. In particular, we illustrate the method’s capacity for missing data imputation, data fusion and clustering.The method can impute data which is missing both *systematically* and *at random*: in a hold-out test on real data, performance is significantly better than commonly used imputation methods. The method’s ability to model inter- and intra-cluster variance leads to more biologically meaningful clusters. The approach removes the necessity for evenly spaced samples, an advantage illustrated on a developmental Drosophila dataset with irregular replications.

**Conclusion:**

The hierarchical Gaussian process model provides an excellent statistical basis for several gene-expression time-series tasks. It has only a few additional parameters over a regular GP, has negligible additional complexity, is easily implemented and can be integrated into several existing algorithms. Our experiments were implemented in python, and are available from the authors’ website: http://staffwww.dcs.shef.ac.uk/people/J.Hensman/.

## Background

Gene expression time course experiments have been used to investigate fundamental biological processes which are often dynamic in nature. For example, the cell cycle [1], cell signalling [2], circadian rhythms [3] and developmental processes [4] have been studied extensively using gene expression time-series data.

Many computational approaches to time-series analysis are not always well suited to gene expression data, where missing measurements are common and time points may not be spaced regularly. In many conventional time-series models such as state-space models [5,6] there is no straightforward manner to deal with missing data, and time points must occur at regular intervals. Whilst gene expression experiments can be sampled regularly, such designs may not be optimal from a statistical or cost perspective. A method for modelling arbitrarily sampled time points may elicit more information from fewer samples, where time points are selected to capture pertinent temporal features.

Furthermore, existing time-series models do not necessarily capture the *structure* of gene expression data. Many gene expression time-series are performed with multiple biological replicates: the crude method of simply averaging the replicates may be discarding interesting information. It is also unclear what to do when the replicates are not sampled at the same times. There is a need for a temporal model which deals with the replicate structure.

Our proposed model is based upon two important ideas: Gaussian process (GP) regression allows for parsimonious temporal inference, whilst a hierarchical structure accounts for (temporally structured) covariance between biological replicates. Additional layers can be added to the hierarchy to model more structure in the data. For example, in a data fusion application, a layer of hierarchy can be used to account for differences between gene expression measurement platforms; or in a clustering application, a hierarchical layer can be added to account for temporal covariance of genes within a cluster.

GPs have been successfully applied to the analysis of gene expression time series by several authors [7-9]. There is little doubt that they provide a coherent and principled framework for regression: for an introduction see [10]. Our contribution is to propose *hierarchical* Gaussian processes to deal with structure in the data. We provide an introduction to the idea, deriving a novel covariance function which accounts for structure. The idea is simple to implement yet highly effective as we demonstrate on several problems. A hierarchical GP model could easily be integrated with existing GP-based applications, allowing them to properly account for replicate structure.

In a further contribution, we manipulate the marginal likelihood expression for the hierarchical GP model for the case where each part of the structure is sampled at the same time, leading to an expression with reduced computational complexity. This situation is most likely to occur during clustering of genes, which must all be measured simultaneously using high throughput methods.

Short time series are prevalent in gene-expression data sets [11]. Our GP-based model is well suited to short time-series, and the behaviour of GPs can be set to mimic that of other temporal models (such as autoregressors) through the covariance function [10], though in this work we use a simple form for the covariance which assures smoothness of the underlying dynamics.

Unlike other time-series based approaches, GPs are not restricted to data which has been sampled at evenly spaced time points. The model therefore removes any restriction on temporal sampling — it can be totally irregular and differ between replicates. This also allows our method to deal with both randomly and systematically missing data. We show how the model can be used for data fusion where the temporal sampling differs between experiments.

## Related work

Hierarchical models are an important idea in Bayesian statistics [12], allowing information to be exchanged between related groups of data. The idea is that by performing inference on the structure as a whole, rather than on each part of the structure independently, inference is improved. GPs have been successfully used in models of gene expression time-series before; for example for inferring transcriptional regulation [8], and to identify differential expression in time-series [7,13]. A key contribution of this work is to combine hierarchical structures with GPs to provide a parsimonious and elegant method for dealing with replicated gene expression time-series.

An alternative to our method was proposed by [14]. In this model, uncertainty is assumed in the *time* of data collection, and the time-shift in each replicate is estimated. In our model, the times are assumed correct whilst the shift is assumed to occur in the expression. Our model has significant computational advantages, since we can marginalise the shifts in expression analytically under the GP framework, whilst the method proposed in [14] required optimisation of a large number of variables (one for each observation). Further, our model is easily included in more complex GP-based models, such as the clustering application which we shall demonstrate. The estimation of time-shifts would be difficult to incorporate into a clustering method, especially considering the very large numbers of parameters which require optimising.

Clustering expression data while modelling within-cluster variance is one of the primary applications of our model. Previously, [15] proposed a random effects model to account for variance between observations of genes and also within clusters of genes. Further, [16] and [17] explored clustering methods with hierarchical structures to model replicate variance. In these models, replicate variance was modelled as multivariate Gaussian around some gene-specific mean, and the gene’s expression was considered multivariate Gaussian around a cluster-specific mean. This paper presents a similar but more powerful idea: we use a hierarchy of GPs to model gene-specific and replicate-specific temporal covariance. We demonstrate that the introduction of a GP prior makes inference of clusters more viable by reducing the number of parameters required to model the data within a cluster, and we also provide a method for dramatically reducing the computational cost of evaluating clusters under our model. Previous methods for clustering temporal data (e.g. [18]) have not used the replicate structure in the model.

## Methods

### Background: Gaussian processes

Gaussian processes (GPs) have been used extensively in a variety of regression problems, and have been applied to gene expression time-series by several authors [7-9]. We briefly introduce GP regression and introduce some notation: for an in-depth introduction one may consult [10].

To perform regression using GPs, we adopt a Bayesian approach. Starting with a prior *directly over functions*, we update the distribution in light of observed data, moving to a posterior distribution. Using standard results for Gaussian distributions, regression involves only some simple linear algebra. The GP prior is fully specified by two functions, a mean function *m*(*t*) and a covariance function *k*(*t*,*t*^′^), and is denoted

(1) $$f(t)∼GP\left(m(t),\;k(t,t^{\prime })\right).$$

For practicality, a zero-mean function is often assumed; throughout this work, the squared-exponential covariance function will be used: *k*(*t*,*t*^′^)=*α* exp{−*γ*(*t*−*t*^′^)^2^}.

**Figure 1:**
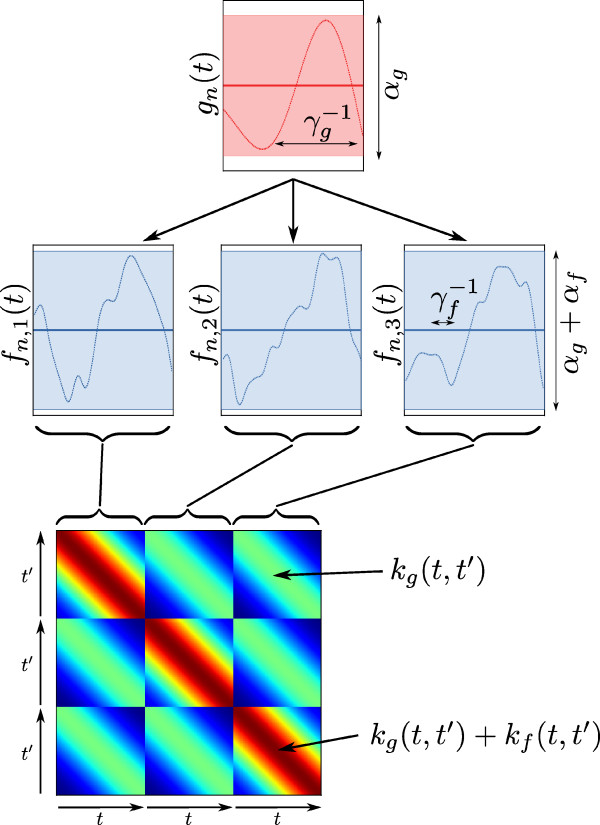
**An illustration of a simple hierarchical GP.** Top: the prior over the underlying function *g*_*n*_(*t*), with the mean *μ*(*t*)=0 shown as a heavy solid line, and the shaded area representing the variance (amplitude) of the function, controlled by the hyper-parameter *α*_*g*_. A single sample from the prior is shown as a narrow line, and the length-scale of the function, inversely controlled by the hyper-parameter *γ*_*g*_, is marked. Middle: three functions, representing three replicates are shown, along with samples conditioned on the sample shown in *g*_*n*_(*t*). The three replicates follow the trend of *g*_*n*_(*t*), but deviate independently by a small amount (variance *α*_*f*_) with a short length-scale, marked in the third replicate. Bottom: the covariance matrix used to generate the samples **Σ**_*n*_. Note the block-wise relationship to the replicates.

Our choice of covariance functions represents a prior belief that the underlying functions are smooth. Other covariance functions could be selected using a model-selection procedure [10]. The parameters of the covariance function (referred to as hyper-parameters) control the amplitude (*α*) and relative length-scale (*γ*) of the functions (see Figure 1 for an illustration). The form of the covariance function captures a very simple assumption about the function: that function points which are close to each other (*t*−*t*^′^ is small) are highly correlated, whilst points which are distant(*t*−*t*^′^ is large) are less correlated.

Regression can be performed by using the marginal and conditional properties of multivariate Gaussian distributions. Supposing we have observations **f** of a function at times **t**, and wish to predict the values of that function at times **t**_⋆_, which we denote **f**_⋆_: the joint probability of **f** and **f**_⋆_ is given by

(2) $$p\left(\left[\begin{array}{c} f\\ f_{⋆} \end{array}\right]\right)=N\left(\left[\begin{array}{c} f\\ f_{⋆} \end{array}\right]\left|\right)0,\left[\begin{array}{cc} K_{t,t} & K_{t,t_{⋆}}\\ K_{t_{⋆},t} & K_{t_{⋆},t_{⋆}} \end{array}\right]\right),$$

where the covariance matrix **K**_**t**,**t**_ has elements derived from the covariance function *k*(*t*,*t*^′^), such that the (*i*,*j*)^th^ element of **K**_**t**,**t**_ is given by *k*(**t**[ *i*],**t**[ *j*]). Consistency of the GP means that it is not necessary to consider the values of the function where we do not have data: these values are trivially marginalised. To perform regression, the conditional property of the multivariate Gaussian gives:

(3) $$p(f_{⋆}|f)=N\left(f_{⋆}|K_{t_{⋆},t}K_{t,t}^{-1}f,\;K_{t_{⋆},t_{⋆}}-K_{t_{⋆},t}K_{t,t}^{-1}K_{t,t_{⋆}}\right).$$

In practice, we are presented with a measurement vector **y** which is a noise corrupted version of **f**. Assuming Gaussian noise^a^ it is possible to write p(y|f)=N(y|f,βI), where *β* is the variance of the noise and **I** the appropriately sized identity matrix, and then marginalise the variable **f**. Equivalently, one can consider **y** to be observations of the Gaussian process *y*(*t*), whose mean function is the Gaussian process *f*(*t*), and covariance function is $\beta \delta_{t,t^{\prime }}$. This hierarchical structure is used later in this publication to build GP priors over replicates and clusters. Either interpretation gives a joint density:

(4) $$p\left(\left[\begin{array}{c} y\\ f_{⋆} \end{array}\right]\right)=N\left(\left[\begin{array}{c} y\\ f_{⋆} \end{array}\right]\left|\right)0,\left[\begin{array}{cc} K_{t,t}+\mathrm{\beta I} & K_{t,t_{⋆}}\\ K_{t_{⋆},t} & K_{t_{⋆},t_{⋆}} \end{array}\right]\right)$$

and regression follows from the conditional property similarly to (3).

Gaussian process regression is a Bayesian method. We move from a *prior* over functions to a *posterior*, and a significant attraction of the method is that this occurs in closed form as (3). However we must still deal with hyper-parameters of the covariance function. Here, we make use of the usual technique which is to optimise the hyper-parameters using type-II maximum likelihood. That is, collecting the hyper-parameters *α*,*β* and *γ* in to a vector ***θ***, we use gradient methods to optimise *p*(**y**|***θ***) with respect to ***θ***. This is given by

(5) $$\begin{array}{c} \log p(y|\theta )=-\frac{D}{2}\log2\pi -\frac{1}{2}\\ \;\times \log|K_{t,t}+\mathrm{\beta I}|-\frac{1}{2}y^{⊤}{\left[\;K_{t,t}+\mathrm{\beta I}\right]}^{-1}y, \end{array}$$

which depends on *θ* through the covariance matrix **K**_**t**,**t**_.

### A hierarchy across replicates

Gene expression time-series may be collected in multiple replicates, to account for biological variation. The idea is that there exists some common trend, present in all replicates, which we wish to identify, and the measurements made of each replicate vary due to biological differences as well as technical noise.

We shall use the notation **y**_*nr*_ to denote the vector of measurements of gene expression of the *n*^th^ gene, in the *r*^th^ biological replicate; these measurements were made at times which we collect into a vector **t**_*nr*_. The data for the *n*^th^ gene is denoted $Y_{n}={\left\{y_{\text{nr}}\right\}}_{r=1}^{N_{n}}$, $T_{n}={\left\{t_{\text{nr}}\right\}}_{r=1}^{N_{n}}$.

Our proposed methodology mimics the structure of the data, directly modelling *underlying* time-series as well as the biological variation, and accounting for (uncorrelated) measurement noise. First consider a time-series model of a single gene. To combine replicates of a particular gene’s time-series, we use a Bayesian hierarchical approach: the underlying expression profile of the *n*^th^ gene *g*_*n*_(*t*) is presumed to be drawn from a zero-mean GP with covariance *k*_*g*_(*t*,*t*^′^), whilst the expression profile of a particular replicate *f*_*nr*_(*t*) is drawn from a GP whose mean is *g*_*n*_(*t*). Thus

(6) $$\begin{array}{c} \begin{array}{cc} g_{n}(t) & ∼GP\left(0,k_{g}(t,t^{\prime })\right),\\ f_{\text{nr}}(t) & ∼GP\left(g_{n}(t),k_{f}(t,t^{\prime })\right). \end{array} \end{array}$$

Note that the two covariance functions *k*_*g*_ and *k*_*f*_ may in general be different: we have used the squared exponential function for both, with independent parameters. This simple model is illustrated in Figure 1, where the dependent nature of the functions is illustrated, as well as the effects of the hyper-parameters.

The elegance of the hierarchical approach lies in its linearity: it is simple to show that two points on the function *f*_*nr*_(*t*) are jointly Gaussian distributed with zero mean and covariance *k*_*g*_(*t*,*t*^′^)+*k*_*f*_(*t*,*t*^′^). Furthermore, two points in separate replicates are jointly distributed with covariance *k*_*g*_(*t*,*t*^′^). Thus, given a set of *N*_*n*_ replicates of gene expression time-series for a particular gene, $Y_{n}={\left\{y_{\text{nr}}\right\}}_{r=1}^{N_{n}}$, taken at different time points $T_{n}={\left\{t_{\text{nr}}\right\}}_{r=1}^{N_{n}}$ it is possible to write the likelihood:

(7) $$p(Y_{n}|T_{n},\theta )=N\left({\hat{y}}_{n}|0,\Sigma_{n}\right),$$

where ${\hat{y}}_{n}$ has been used to denote the concatenation of **Y**_*n*_, ${\hat{y}}_{n}={\left[\;y_{n,1}^{⊤},y_{n,2}^{⊤}\ldots y_{n,N_{n}}^{⊤}\right]}^{⊤}$, *θ* represents the parameters of the covariance functions *k*_*g*_ and *k*_*f*_, and the block of **Σ**_*n*_ corresponding to $y_{\text{nr}},y_{nr^{\prime }}$ is given by

(8) $$\;\Sigma_{n}[\;r,r^{\prime }]=\left\{\begin{array}{cc} K_{g}(t_{\text{nr}},t_{nr^{\prime }})+K_{f}(t_{\text{nr}},t_{nr^{\prime }})+\mathrm{\beta I} & \text{if}\;r=r^{\prime }\\ \;K_{g}(t_{\text{nr}},t_{nr^{\prime }})\;\text{otherwise.} \end{array}\right)$$

In order to make inferences about the functions *g*_*n*_(*t*) and *f*_*nr*_(*t*), the covariances between the data **Y** and the functions are required. Using the superscripted **y***nr*(*t*) to denote the element of **y**_*nr*_ observed at time *t*:

(9) $$\text{cov}\left(y_{\text{nr}}^{\left(t\right)},g_{n}(t^{\prime })\right)=k_{g}(t,t^{\prime }),$$

(10) $$\text{cov}\left(y_{\text{nr}}^{\left(t\right)},f_{nr^{\prime }}(t^{\prime })\right)=\left\{\begin{array}{cc} k_{g}(t,t^{\prime })+k_{f}(t,t^{\prime }) & \text{if}\;r=r^{\prime }\\ \;k_{g}(t,t^{\prime })\;\text{otherwise.} \end{array}\right)$$

Inferences about functions can then be made using the standard methods described above, and hyper-parameters of the covariance functions can be optimised.

**Figure 2:**
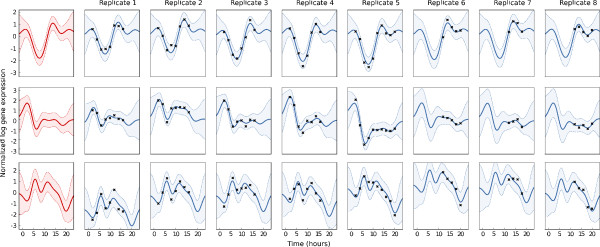
**Hierarchical GP regression on the expression of three illustrative genes during Drosophila Melanogaster development, using eight replicates with different time sampling.** Each row represents one gene. The leftmost panes show the inferred function *g*_*n*_(*t*), subsequent panes represent the replicates. Solid lines show the mean of the predicted function, and the shaded area represents the 95% confidence interval. The parameters of the covariance function were optimised by maximum likelihood. Note that the replicates contain different numbers of data and different times.

Fitting a hierarchical model to a set of replicates can be used as a diagnostic tool. In particular, by examining the maximum-likelihood values of the covariance function parameters, we can assess how noisy the experiment is, and how similar the replicates are. Figure 2 shows three examples of hierarchical regression of the time course data described the Results section, for three genes (modelled independently), with each gene shown in one row in the figure. The leftmost panes in the figure show the inferred function for the gene, *g*_*n*_(*t*), and subsequent panes show the inferred functions for each replicate, *f*_*nr*_(*t*).

These examples demonstrate different behaviours of the time-series which are captured by the model. For the first gene, CG18135, the replicates are quite similar, and most deviation of the data from *g*_*n*_(*t*) is attributed to noise. The model attributes 87% of the data’s variation to the underlying function *g*_*n*_(*t*), and only 6% each to replicate variance and noise, i.e. the model ‘recognised’ the similarity of the replicates.

The gene represented by the middle row is AP-47, and it can be seen that there is considerable replicate variance: although each replicate follows a similar pattern, the pattern is ‘amplified’ differently in the replicates. Here, the model attributes 60% of the data’s variance to the function *g*_*n*_(*t*) and 34% to *f*_*nr*_(*t*), with 6% to noise.

The gene represented by the bottom row of Figure 2 is OstStt3. Here, the variances of *g*_*n*_(*t*), *f*_*nr*_(*t*) and noise are 55%, 36% and 8% respectively. The model recognises the differences in the replicates, but uses a long length scale for *f*_*nr*_(*t*). In this gene, the detailed pattern of the time-series is captured entirely by *g*_*n*_(*t*), and *f*_*nr*_(*t*) is used to account for amplitude shifts between replicates. Note that these cannot be simply ‘normalised out’ because not all replicates cover the same temporal region. These genes were selected using a simple filtering procedure. The model was fitted independently to each gene on a microarray, and the genes were ranked according to the ratio of signal variance (a hyperparameter of *k*_*g*_) and replicate-plus-noise variance (hyperparameters from *k*_*f*_).

### Deeper hierarchies

In many cases, gene expression time-series may have more structure than simply biological replicates. For example, we could incorporate previous studies in a hierarchical fashion. In general, suppose that there is some underlying function *g*_*n*_(*t*) which models the general gene expression activity for the *n*^th^ gene. Subsequently, we define the functions *e*_*ni*_(*t*) for each experiment which we want to model, and finally *f*_*nir*_(*t*) for the r ^th^ replicate in the i ^th^ experiment.

(11) $$\begin{array}{cc} g_{n}(t) & ∼GP\left(0,k_{g}(t,t^{\prime })\right),\\ e_{\text{ni}}(t) & ∼GP\left(g_{n}(t),k_{e}(t,t^{\prime })\right),\\ f_{\text{nir}}(t) & ∼GP\left(e_{\text{ni}},k_{f}(t,t^{\prime })\right). \end{array}$$

With every layer of the hierarchy, we have introduced new parameters corresponding to the covariance function for that layer. Note that the hierarchy can be extended arbitrarily to represent the structure of the data. For example, we might want to model biological variation where the lineage is known, or to model inter-species variation, or to build a hierarchy which reflects the phylogenetic relationship between species.

### An efficient model of clusters

Clustering of gene expression time-series is often performed with a view to finding groups of co-regulated or associated genes. The central assumption is that genes which are involved in the same biological processes will be expressed together: they share an underlying time-series.

In order to model a group of genes as defined by a cluster, the hierarchical model is extended to a three-layer hierarchy across the cluster, individual genes and replicates.

All genes in the *i*^th^ cluster are presumed to share an underlying profile *h*_*i*_(*t*), and subsequently each gene follows a profile *g*_*n*_(*t*) and each replicate of that gene follows a profile *f*_*nr*_(*t*). The mean of each level in the hierarchy is given by the level above, so the data **Y**_*i*_ in cluster *i* is modelled by:

(12) $$\begin{array}{cc} h_{i}(t) & ∼GP\left(\text{0},k_{h}(t,t^{\prime })\right),\\ g_{n}(t) & ∼GP\left(h_{i}(t),k_{g}(t,t^{\prime })\right),\\ f_{\text{nr}}(t) & ∼GP\left(g_{n}(t),k_{f}(t,t^{\prime })\right). \end{array}$$

If ${\hat{y}}_{i}$ is the concatenation of all of the ${\hat{y}}_{n}$ representing genes in the *i*^th^ cluster, noting that each ${\hat{y}}_{n}$ is itself a concatenation of the biological replicates, then the marginal likelihood of the expression data in the *i*^th^ cluster, **Y**_*i*_ is given by

(13) $$p(Y_{i}|T_{i})=N\left({\hat{y}}_{i}|0,\;\Sigma_{i}\right)$$

where the covariance matrix **Σ**_*i*_ is structured such that the block corresponding to the two genes *n* and *n*^′^ is given by

(14) $$\Sigma_{i}[\;n,n^{\prime }]=\left\{\begin{array}{cc} \Sigma_{n}+K_{h}(t_{n},t_{n}) & \text{if}\;n=n^{\prime }\\ \;K_{h}(t_{n},t_{n^{\prime }}) & \text{otherwise.} \end{array}\right)$$

Note that the diagonal blocks of **Σ**_*i*_ are *themselves* block-structured, reflecting the double hierarchy in the model.

The computational complexity of this model grows cubically as the size of the cluster increases, which is an undesirable property. To reduce the computational load, it is possible to exploit a known property of the data. In each array all genes are simultaneously measured, although we allow different times for each replicate. Denote $\hat{t}$ the concatenation of the times in all replicates, define **K**_*h*_ as the covariance matrix formed by evaluating *k*_*h*_ on the grid of $\hat{t}$, and **Σ**_*n*_ a covariance matrix structured as (8), modeling the variance of a single gene. The marginal likelihood can then be written

(15) $$\begin{array}{c} p(Y_{i}|T_{i})=2\pi^{-\frac{N_{i}D}{2}}|K_{h}{|}^{\frac{1}{2}}|\Sigma_{n}{|}^{\frac{N_{i}}{2}}|K_{h}^{-1}+N_{i}\Sigma_{n}{|}^{\frac{1}{2}}\\ \;\exp\{-\frac{1}{2}\;\{\overset{N_{i}}{\underset{n=1}{\sum }}\;\overset{⊤}{\underset{n}{y}}\;\overset{-1}{\underset{n}{\Sigma }}y_{n}\;-\;\overset{2}{\underset{i}{N}}\overset{⊤}{\underset{i}{\bar{y}}}\;\overset{-1}{\underset{n}{\Sigma }}{\left(\overset{-1}{\underset{h}{K}}\;+\;\overset{-1}{\underset{n}{\Sigma }}\right)}^{-1}\overset{-1}{\underset{n}{\Sigma }}{\bar{y}}_{i}\}\}, \end{array}$$

where ${\bar{y}}_{i}$ is the mean of the ${\hat{y}}_{n}$ in the cluster, *D* is the length of $\hat{t}$, and *N*_*i*_ is the number of genes in the cluster (see appendix for a derivation). This expression has reduced the computational complexity of the model from $O(N_{i}^{3}D^{3})$ to $O(D^{3})$.

**Figure 3:**
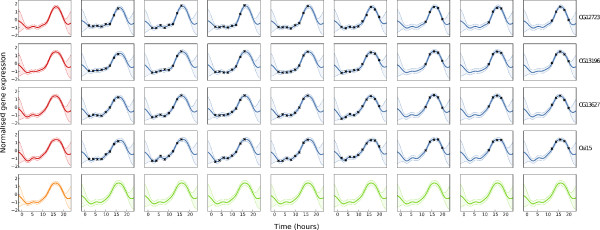
**A hierarchical model of expression across multiple genes within a cluster.** Each row represents one gene (gene names are to the right) and each box within that row represents a biological replicate. Data are represented as black points, the shaded area represents 95% confidence interval and a solid line represents a posterior mean function. The left-most box on each row shows the inferred function for each gene *g*_*n*_(*t*), and the bottom row shows the mean function for the cluster *h*(*t*) (left) and the predicted function(s) for a hypothetical new gene.

An example of this model is shown in Figure 3. The inferred function *h*(*t*), shown in the bottom-left pane has a single wide peak at around 15 hours; all of the functions *g*_*n*_(*t*) (leftmost column) show a similar pattern, though the functions are each ‘distorted’ a little, with the width of the peak varying from gene to gene. Similarly, each replicate shows a similar pattern to the mean function for the corresponding gene, with smaller variations. The bottom row shows the predictive density for a new gene within the cluster.

### Clustering

To use our model for clustering, the partitioning of genes into clusters needs to be inferred. Dunson [18] proposed a clustering scheme where a GP is used to model the function within a cluster, and a Dirichlet process prior is placed on the partitioning. This leads to a Gibbs sampling scheme where each Gibbs step involves removing a gene from the clustering and then stochastically re-allocating it. Our model potentially improves on Dunson’s formulation since we consider a structured covariance across the genes and replicates (which was treated as iid noise by Dunson), though it would be possible to use the same Gibbs sampling scheme to infer the cluster partitions.

Heller and Ghahramani [19] showed that inference in a DP can be effectively approximated using an agglomerative clustering scheme dubbed Bayesian Hierarchical Clustering (BHC)^b^. Cooke et al. [20] applied this hierarchical clustering scheme to gene expression time-series with a GP prior, and we extend their work using a hierarchically structured GP to model the clusters, as well as the efficient computation of the marginal likelihood as per equation (15).

The algorithm is depicted in Algorithm 1, and works in a ‘bottom-up’ fashion. Starting with each gene in an individual cluster, the clusters are merged by greedily selecting the merge which maximises the log marginal likelihood of the data (by summing the log marginal likelihood over all clusters). Once no more merges are available to improve the overall marginal likelihood, the hyper-parameters are optimised, and the procedure is repeated with the new covariance function in an EM fashion.

## Results and discussion

In a recent study of *Drosophila* development [21], gene expression was measured in eight replicates measured across six species at differing time-points. 3695 genes (with orthologs across the species) were investigated using Agilent microarrays. Here we focus on Melanogaster development: time courses for typical genes are shown in Figure 2 and 3, with eight replicates at up to thirteen time-points. Note in particular that no replicate contains all the time-points: some replicates cover only the last few points, whilst some have broader coverage.

For all the missing data experiments, the covariance function hyper-parameters were set to the maximum likelihood value using gradient based-numerical optimisation. Whilst we show that the hierarchical GP has better performance than the GP in all cases, it does not require any extra computation. All experiments took only a few minutes on a desktop PC.

### Missing data imputation

The imputation of missing data is a straightforward method for validation of our model. In this Section, we remove data *systematically*, effectively removing entire microarrays from the experiment and predicting what was on them. Most missing data imputation methods cannot handle this type of missing data, highlighting an advantage of our method. This experiment also validates our assertion that it is important to include the replicate structure in modelling microarray time-series, and that simply averaging the data on a time-point basis is not satisfactory.

**Algorithm 1** Cluster replicated gene expression time-series 

Whilst systematically missing data are not common in the laboratory, this test does examine the performance of our model in some potentially interesting applications. For example, we may wish to predict the future gene expression levels of a patient given the time series observed in other patients.

The results of imputing missing data are compared with the simple but oft-used technique of averaging the replicates, using both the mean and median of the non-missing replicates. The method is also compared with a simple GP model which does not account for replicate structure. We investigated the effectiveness of our algorithm using varying amounts of missing data, removing 1, 5, 10 and then 20 of the 56 microarrays at random. Each experiment was repeated 10 times with different randomisations; for each we computed the RMSE (root mean square error) averaged over all missing arrays and over all genes. The mean RMSE and two standard deviations as measured over the randomisations are shown in Table 1.

**Table 1:** RMSE for missing data imputation for differing numbers of randomly removed arrays

	**1 of 56**	**5 of 56**	**10 of 56**	**20 of 56**
HGP	**0 *. *30±0 *. *27**	**0 *. *32±0 *. *09**	**0 *. *34±0 *. *05**	**0 *. *38±0 *. *06**
GP	0.46±0.23	0.44±0.09	0.43±0.08	0.46±0.07
Mean	0.52±0.24	0.48±0.12	0.48±0.11	0.48±0.08
Median	0.50±0.25	0.46±0.11	0.47±0.11	0.48±0.08

The rows correspond to Hierarchical GPs (HGP), non-hierarchical GPs (GP), and imputation using the mean and median of available replicates. Columns represent increasing amounts of missing data.

Table 1 shows that the hierarchical GP performs better at imputing the missing data in all examined cases. Although the Table shows only the average over randomisations, the HGP algorithm gave the lowest RMSE for every randomisation that we tried. The standard deviations in the Table generally decrease as the number of missing points increases. This reflects the degree to which the missing data imputation depends on which time-points are missing, which may be due to the different temporal sampling schemes employed in the different replicates.

We note from Table 1 that our contribution of adding replicate structure to the GP methodology makes a significant difference to the results, since the standard GP offers only modest improvement over the simple averaging methods. We also note that the averaging methods are only possible where time-points are duplicated between replicates, a restriction which the (H)GP methodology removes.

### Randomly missing data imputation

Our proposed model is novel in the sense that it can impute entire missing arrays, as above. Most missing data algorithms assume randomly missing data and use correlation between genes for imputation. To compare our algorithm with those from the literature, we randomly removed 100 values from the *Melanogaster* dataset, and measured the error on imputation. For comparison, we also used two popular methods, K-nearest-neighbour (KNN) [22] and Bayesian principal component analysis (PCA) [23].

**Figure 4:**
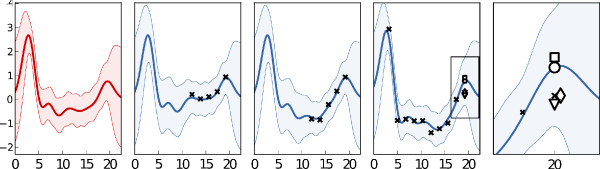
**An example of randomly missing data imputation.** Here, the left-most pane shows the inferred mean function for the gene *g*_*n*_(*t*), and the next three panes show three replications, in the last of which the missing data imputation occurs. Other replications are omitted for brevity. The final panel shows a zoom of the imputed region (boxed in the previous panel). Imputation by the HGP is marked by a circle on the mean function; replicate median is denoted by a square; PCA is denoted by a diamond and KNN by a triangle. The HGP imputation is closer to the replicable statistic, being less affected by cross-sectional noise effects than the other methods.

Gene expression experiments usually contain many types of effect aside from the one under study. In this case, the data includes cross-sectional effects which arise from array-specific and sample-specific causes, and are not due to the underlying time-series. These are treated as noise by our model, whereas PCA and KNN make no distinction between longitudinal and cross-sectional variance and will freely impute these effects. This is illustrated in Figure 4, where cross-sectional effects mean that the missing datum’s true value lies below that seen by averaging the replicates, or imputed by HGP. The HGP and KNN methods, being sensitive to these effects impute the true value well, despite it being inconsistent across replicates.

To test the methods’ abilities to impute the replicable part of the signal, we tested the imputed values of the three methods against the median value for the missing time-point, averaged across replicates. We measured the RMSE over the 100 imputations, and repeated the experiment 10 times with different randomisations. The mean RMSE (over randomisations) and two standard deviations are shown in the first column of Table 2.

**Table 2:** RMSE of missing data imputation for hierarchical Gaussian processes (HGP) principal component analysis (PCA) and K-nearest neighbour (KNN)

	**Replicate median**	**Full model**
HGP	**0 *. *13±0 *. *03**	**0 *. *05±0 *. *01**
PCA	0.15±0.03	0.10±0.02
KNN	0.17±0.04	0.13±0.02

The first column shows the RMSE measured against the replicate-median, and the second column shows the RMSE against the model with no missing data.

Another way to investigate the ability of the model to deal with missing data is to examine the difference between the model as inferred with some missing points to that inferred with all the data: the results of doing so are shown in the second column of Table 2. Whilst this method may not give a completely fair reflection of the performance of the PCA and KNN methods, the small size of the errors on imputation imply that our model is relatively *insensitive* to missing data: because the model can borrow statistical strength from other replicates, small amounts of data missing at random make little difference to the model.

### Data fusion

The data under investigation are sampled at two-hour intervals. To improve our knowledge of the system, it is possible to perform data fusion with existing data sets. We demonstrate this with two previous studies: [4,24], which offer more tightly temporally-spaced data, but with fewer replications (three and one respectively).

**Figure 5:**
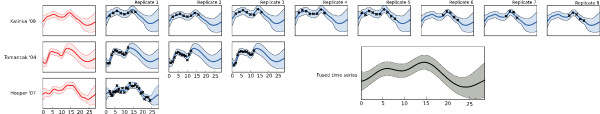
**Hierarchical GP regression on across three data sets, for the gene Acer.** Each data set is represented by one row, and each replicate within a data set is represented by a single pane in that row. Shaded regions represent 95% confidence intervals. Inset: the fused time-series. (Y-scales removed for clarity but are consistent between plots).

We constructed a hierarchical model across the three experiments, and across replicates within the experiments. The data were considered on a gene-by-gene basis, and the model was optimised for each gene. An example gene (Acer) is shown in Figure 5. The figure shows the inferred function for each replicate in each experiment, as well as the inferred mean function for each experiment (first column) and the inferred ‘top-level’ function (inset) which underlies all the experiments.

### Clustering

In order to investigate the usefulness of our model in a clustering task, we first selected 300 dynamically differentially expressed genes using a method similar to [7].

We computed the marginal likelihood using our hierarchical model and a simpler GP model *without* replicate-specific or gene-specific variance. This model, simply fitting a GP to the lumped data is similar to the method proposed in [20], which represents the current state-of-the-art. Cooke *et al*[20] compared their method to several other algorithms and concluded that the GP method allowed for the discovery of clusters in a more effective manner than non-temporal models. Here we show that this method can be improved further by considering the gene-wise and replicate-wise intra-cluster structure of the data’s variance. For both models, we applied the EM algorithm with several restarts (varying the covariance function hyper-parameters on each restart), selecting the solution with the highest log-likelihood. We used our own implementation for Cooke et al.’s method to ensure that results were comparable: i.e. that improvements in the method were due to the HGP structure, rather than specifics of the implementation. We are primarily concerned with the improvements that the HGP model can offer in explaining cluster variance, and this allows for a direct comparison.

For further comparison, we used the R package Mclust [25]. Mclust fits a range of Gaussian models of increasing complexity; in the first instance, we simply concatenated the replicates and Mclust struggled to fit some models in the 56 dimensional space. Subsequently, we provided Mclust with shorter vectors formed by averaging the replicates at each time point, which gave similar results. We include the validation for both methods. In both cases, we used all available covariance structures for Mclust, and let the package pick the best using its BIC (Bayesian Information Criterion) approach.

In order to validate the different clusterings, we use the biological homogeneity index (BHI) [26], which assesses the number of genes with common function within each cluster, assigning the entire clustering a score from 0 to 1 with larger values corresponding to more biologically homogeneous clusters. For biological annotation, we used the three gene ontology (GO) categories (data obtained using biomaRt [27]), which correspond to molecular function (MF), biological process (BP) and cellular component (CC). Computation of the BHI is then straightforward: it is the proportion of within-cluster pairs that share at least one biological annotation. The BHIs and log-likelihoods for all the experiments are shown in Table 3. We note that other comparisons between the clusters and the Gene Ontology may be possible, for example the GO term overlap score [28,29], but we use the BHI here for ease of interpretability.

**Table 3:** Results of clustering 300 genes using the four proposed algorithms

	**MF**	**BP**	**CC**	ℒ	**N. clust.**
Agglomerative HGP	**0 *. *92**	**0 *. *32**	**1 *. *0**	**7360 *. *8**	50
agg. GP (as Cooke *et al.*)	0.78	0.26	0.72	6203.7	128
Mclust (concat.)	0.78	0.14	0.50	1324.0	26
Mclust (averaged)	0.80	0.16	0.42	-736.2	20

The first three columns report the Biological Homogeneity Index (BHI) for the gene ontology categories of Molecular Function (MF), Biological Process (BP) and Cellular component (CC). The two last columns report the log-likelihood ℒ of the models and the number of recovered clusters.

From the Table, it can be seen that HGP method improves the biological homogeneity for all three GO categories. By directly comparing with the standard GP method, we have demonstrated that the improvement in clustering performance is not due simply to the clustering methodology or the GP correlations which give the GP method a small improvement over Mclust, but the hierarchical structure of intra-cluster variance which allows genes and replicates to differ in a temporally-correlated fashion.

## Conclusions

We have presented a method based on hierarchically structured GPs, which are a practical and flexible framework for modelling replicated time-series. The framework has a wide range of applications, and can be extended for various data structures besides biological replications.

The method performed well in several tasks, including missing data imputation and clustering. We have shown that the method performs particularly well in missing data imputation, and that small amounts of missing randomly data have only a minor effect of the model. Biological validation through the BHI confirms the importance of modelling intra-cluster variance in a hierarchical fashion.

Above we showed how fitting the simplest of our proposed models can lead to a quantitative assessment of how biological replications are behaving, as well as illustrating how our method deals with different types of replicate variation. Of course, if the replicate variance is truly independent – e.g. if only technical variation is present – then we recover standard GP regression. In this case the hierarchical approach requires the inclusion of an extra parameter, but we find that the additional computational complexity is negligible.

A problem with standard GP regression is that the computational complexity grows cubically with the number of data. We have presented a method which exploits the necessary condition that all genes in a cluster are measured on the same time-points in order to significantly reduce the computational complexity and make our clustering algorithm applicable to large data sets. We note that the complexity of the clustering algorithm scales poorly with the number of genes: the initial step of the algorithm must compare the likelihood of merging of each pair. To address this, randomised versions of the same algorithm can be adopted [30,31], and our hierarchical model and its efficient computation as (15) could be used with no modification.

Whilst we have demonstrated that our model is useful in several applications, we envisage a number of extensions. For example, our model could be used for data fusion of microarray data with high-throughput sequencing data. Or, the hierarchical structure could be used in models of pathway activity [32], which may include prior information about gene groupings from Gene Ontology.

Although we have only used simple GP models within our hierarchical structure, the idea can be applied to more complex GP models, such as those proposed to model gene interactions [9,33].

## Endnotes

^a^ Other noise distributions are possible, but break the conjugacy of the model and thus complicate inference, see [10].

^b^ Note that hierarchical in this sense means a hierarchical partitioning of the genes, distinct from our Bayesian hierarchical model applied within the cluster.

## Appendix

### Efficient computation of a cluster likelihood

The expression for the marginal likelihood of a cluster of genes as given in equation (15) can be derived by considering the values of the underlying function *h*(*t*) at the time points **t**, which we denote **h**. The model (for a single cluster) can be written:

(16) $$p(Y_{i}|T_{i})=\int p(h|t_{⋆})\underset{k\in c_{i}}{\prod }p(y_{k}|h,t_{⋆})\;\text{d}h.$$

This consists of a prior for the latent variable **h** and a likelihood for the data associated with each gene in the cluster, conditioned on the latent variable. The objective here is to marginalise (integrate-out) the latent variable to achieve a tractable expression. Expanding equation (16),

(17) $$\begin{array}{c} p(Y_{i}|T_{i})=\int {\left(2\pi \right)}^{-(N+1)D/2}|\Sigma_{n}{|}^{-N/2}|K_{h}{|}^{-1/2}\\ \;\exp\left\{-\frac{1}{2}h^{⊤}K_{h}^{-1}h\right)\\ \left(\;-\frac{1}{2}\underset{k\in c_{i}}{\sum }{\left({\hat{y}}_{k}-h\right)}^{⊤}\Sigma_{n}^{-1}({\hat{y}}_{k}-h)\right\}\;\text{d}h. \end{array}$$

Some re-arrangement and completing the square inside the exponent leads to

(18) $$\begin{array}{c} p(Y_{i}|T_{i})=\int {\left(2\pi \right)}^{-(N+1)D/2}|\Sigma_{n}{|}^{-N/2}|K_{h}{|}^{-1/2}\\ \exp\left\{-\frac{1}{2}{\left(h-\hat{h}\right)}^{⊤}\Lambda (h-\hat{h})\right)\\ \left(\;\;-\frac{1}{2}{\hat{h}}^{⊤}\Lambda \hat{h}-\frac{1}{2}\underset{k\in c_{i}}{\sum }{\hat{y}}_{k}^{⊤}\Sigma_{n}^{-1}{\hat{y}}_{k}\right\}\;\text{d}h. \end{array}$$

where we have defined for brevity $\Lambda \;=\;K_{h}^{-1}\;+\;N_{g}\;\Sigma_{n}^{-1}$ and $\hat{h}=\Lambda^{-1}\Sigma^{-1}N_{g}{\bar{y}}_{i}$. The first and third lines of this expression can be moved outside the integral, and we recognise the Gaussian nature of $\int \;\exp\left\{-\frac{1}{2}{\left(\text{h}-\hat{h}\right)}^{⊤}\Lambda (h-\hat{h})\right\}\;\text{d}h={\left(2\pi \right)}^{D/2}|\Lambda {|}^{1/2}$. Substituting this and the expressions for $\hat{h}$ and ***Λ*** back into (18) leads to the expression given in (15).

## Competing interests

The authors declare that they have no competing interests.

## Authors’ contributions

JH designed the studies, implemented the algorithms in python and drafted the manuscript. MR and NDL assisted in design and analysis of the experiments. All authors read and approved the final manuscript.
